# Social Media Recruitment Strategies to Recruit Pregnant Women Into a Longitudinal Observational Cohort Study: Usability Study

**DOI:** 10.2196/40298

**Published:** 2022-12-12

**Authors:** Chloe Pekarsky, Janice Skiffington, Lara M Leijser, Donna Slater, Amy Metcalfe

**Affiliations:** 1 Department of Obstetrics and Gynecology University of Calgary Calgary, AB Canada; 2 Section of Neonatology Department of Pediatrics University of Calgary Calgary, AB Canada; 3 Department of Physiology and Pharmacology University of Calgary Calgary, AB Canada; 4 Departments of Community Health Sciences and Medicine University of Calgary Calgary, AB Canada

**Keywords:** social media, Facebook, Twitter, Instagram, recruitment, pregnancy, surveys, questionnaires, fraudulent responses

## Abstract

**Background:**

Use of social media for study recruitment is becoming increasingly common. Previous studies have typically focused on using Facebook; however, there are limited data to support the use of other social media platforms for participant recruitment, notably in the context of a pregnancy study.

**Objective:**

Our study aimed to evaluate the effectiveness of Facebook, Twitter, and Instagram in recruiting a representative sample of pregnant women in a longitudinal pregnancy cohort study in Calgary, Alberta, between September 27, 2021, and April 24, 2022.

**Methods:**

Paid advertisements were targeted at 18- to 50-year-old women in Calgary, with interests in pregnancy. Data regarding reach, link clicks, and costs were collected through Facebook Ads Manager (Meta Platforms, Inc) and Twitter Analytics (Twitter, Inc). The feasibility of each platform for recruitment was assessed based on the recruitment rate and cost-effectiveness. The demographic characteristics of the participants recruited through each source were compared using the chi-square test.

**Results:**

Paid advertisements reached 159,778 social media users, resulting in 2390 link clicks and 324 participants being recruited. Facebook reached and recruited the highest number of participants (153/324, 47.2%), whereas Instagram saw the highest number of link clicks relative to the number of users who saw the advertisement (418/19,764, 2.11%). Facebook and Instagram advertisements were cost-effective, with an average cost-per-click of CAD $0.65 (US $0.84; SD $0.27, US $0.35) and cost-per-completer of CAD $7.89 (US $10.25; SD CAD $4.08, US $5.30). Twitter advertisements were less successful in terms of recruitment and costs. Demographic characteristics of participants did not differ based on recruitment source, except for education and income, where more highly educated and higher-income participants were recruited through Instagram or Twitter. Many issues related to fraudulent responses were encountered throughout the recruitment period.

**Conclusions:**

Paid social media advertisements (especially Facebook and Instagram) are feasible and cost-effective methods for recruiting a large sample of pregnant women for survey-based research. However, future research should be aware of the potential for fraudulent responses when using social media for recruitment and consider strategies to mitigate this problem.

## Introduction

### Background

Recruitment is crucial to the success of prospective studies; however, it remains one of the most challenging aspects of conducting research owing to its time-consuming and expensive nature [1]. To address the challenges of recruitment, researchers are increasingly turning to social media as a tool to reach potential participants [1,2]. Using social media for recruitment is particularly beneficial in the context of the COVID-19 pandemic where many health care services have moved to web-based formats, thus decreasing the likelihood that participants encounter study advertisements through traditional methods such as posters or postcards.

Approximately 85% of Canadians reported using social media in the past year, with Facebook, Twitter, and Instagram being 3 of the 4 most commonly used platforms [1,3]. Women between the ages of 25 and 34 years account for the greatest proportion of social media users in the country [3], demonstrating the potential of social media to reach pregnant women for study recruitment. Previous studies focusing on pregnancy have found that using traditional recruitment methods in conjunction with paid social media advertisements is an effective way of recruiting desired number of participants within a short period [1,4-6]. However, most literature on this topic focuses exclusively on comparing traditional methods with Facebook. Therefore, it is unknown whether findings from Facebook are generalizable to other platforms such as Twitter and Instagram.

### Objectives

By examining the success of multiple paid advertisement campaigns across Facebook, Instagram, and Twitter simultaneously, this study aimed to determine (1) which social media platform is most effective for recruitment in terms of recruitment rate and cost; (2) what kind of advertisements lead to the most engagement with our study and the most number of participants; and (3) whether participants recruited through each platform differed from each other and from participants recruited through traditional methods.

## Methods

### The P3 Cohort Study

The P3 Cohort Study (Prediction, Prevention and Interventions for Preterm Birth) is a longitudinal cohort study aimed at recruiting 4000 pregnant women and their partners in Calgary, Alberta, to better understand preterm birth [7]. The study comprises 5 web-based surveys to be completed during pregnancy and the first year postpartum. In addition, the partner may choose to participate in 2 surveys. As of April 2022, participant pregnancy status and identity are self-reported but, following completion of recruitment, will be verified by medical records. Participants are compensated with a CAD $10 (US $13) electronic gift card for every survey they complete. Participants are eligible for this study if they are <32 weeks pregnant with a singleton pregnancy, living in the Calgary Zone of Alberta Health Services, and ≥16 years old.

### Recruitment Platforms and Study Advertisements

Beginning in September 2021, paid advertisements targeting women between the ages of 18 and 50 years living within a 20-mile radius of Calgary with specific interests in pregnancy and parenting were used to facilitate recruitment. Facebook- and Instagram-specific targeting features included “motherhood or baby shower and parents: parents (all),” and Twitter-specific targeting features included “family and parenting- babies and toddlers, family and parenting- daycare and preschool, family and parenting- parenting K-6 kids, life stages- moms.” The terms of service of the social media platforms did not allow us to use these specific targeting features for users <18 years of age. For nearly every advertisement campaign, the appearance of study advertisements included (1) a title (ie, “Are you less than 32 weeks pregnant?”), (2) a description (ie, “Help UCalgary researchers and join our study to understand preterm birth!”), (3) an image or a graphic (ie, a pregnant person and a baby in the neonatal intensive care unit), (4) institutional logos to establish the credibility of the study (ie, the University of Calgary, the Calgary Health Foundation, and the Alberta Children’s Hospital Foundation), and (5) a link to our website.

Between September 27, 2021, and April 24, 2022, a total of 13 campaigns were run, with each advertisement being manipulated in terms of budget, duration, and content (Table 1). Each campaign involved the same advertisement running on multiple social media platforms simultaneously. Most of the advertisements contained only the essential information, while other advertisements were themed (eg, Halloween and Prematurity Awareness Month). In addition, some advertisements mentioned the study incentive of CAD $10 (US $13; although participants received the incentive for each survey completed regardless of whether or not this was mentioned in the advertisement). During this 7-month recruitment period, traditional methods (eg, posters and postcards) were distributed in the community and through health care providers to potential participants.

**Table 1:** Duration, budget, and image for each of the 13 advertisement campaigns used throughout recruitment.

Campaign	Duration, day	Budget (per day), CAD $ (US $)	Image
0^a^	N/A^b^	N/A	Standard^c^
1^a^	N/A	N/A	Standard
2	5	25 (32.5)	Standard
3	3	10 (13)	Halloween^d^
4	5	10 (13)	Prematurity Awareness Month^e^
5	5	35 (45.5)	Standard
6	5	50 (65)	Standard
7	3	25 (32.5)	Standard
8	3	25 (32.5)	Incentive mentioned^f^
9	5	25 (32.5)	Incentive mentioned
10	5	50 (65)	Incentive mentioned
11	8	25 (32.5)	Incentive mentioned
12	1^g^	35 (45.5)	Incentive mentioned

^a^Campaigns 0 and 1 were unpaid to address the issues associated with fraudulent participants. Unpaid advertisements are not boosted by the social media platforms and are consequently shown to fewer users. These campaigns can still mention the incentive as participants who see the advertisement will still be compensated for their participation. Campaign 0 mentioned the incentive in the caption of the campaign but not in its image.

^b^N/A: not applicable.

^c^The standard advertisement refers to a post that included a title (“Less than 32 weeks pregnant?”), a brief description of the study, our study website, a relevant cartoon, and logos of affiliated institutions. There was no mention of incentives.

^d^The Halloween advertisements compared the size of a baby at different gestational age to the Halloween candy. There were no logos of the institutions with which we were affiliated and no mention of the incentive. Information regarding the study was provided in the caption.

^e^The Prematurity Awareness Month advertisement was posted in November and included a title (“November is Prematurity Awareness Month”), an image of an infant in the neonatal intensive care unit, and the logos of the affiliated institutions. There was no mention of any incentives.

^f^The incentive advertisements were the same as the standard advertisement but mention the incentive directly in the photo of the advertisement.

^g^Campaign 12 was scheduled to run for 5 days but was discontinued after only a day owing to a high number of fraudulent responses.

### Data Collection

Data regarding reach, link clicks, and cost for each campaign were collected through Facebook Ads Manager (which includes data for both Facebook and Instagram) and Twitter Analytics (Textbox 1 for definitions). Data regarding where participants learned about the study (Facebook, Twitter, Instagram, or a traditional source) and demographic data, including age, education, income, race, country of birth, and marital status, were obtained from the baseline survey to determine whether demographic characteristics varied between recruitment methods.

**Definitions of study outcomes.**

**Reach**

- Number of users who saw the advertisement on their feed

**Link clicks**

- Number of users who clicked on the advertisement link

**Click-through-rate**

- Number of link clicks divided by reach

**Completion to click ratio**

- Number of completers divided by number of link clicks

**Cost-per-click**

- Total cost of the advertisement divided by the number of link clicks for each advertisement

**Cost-per-completer**

- Total cost of the advertisement divided by the number of people who completed the baseline survey

### Program Evaluation Method

The effectiveness of Facebook, Twitter, and Instagram was evaluated based on the recruitment rate and cost-effectiveness. The analysis was broken down by each advertisement campaign, which spanned the day the campaign started to the day before the next campaign. The number of participants who consented to each campaign was used, as it was assumed that the consent date would more accurately reflect the day that the participant saw the study advertisement compared with the date that they completed the baseline survey (eg, participants could have consented days before completing the baseline survey).

#### Recruitment Rate

The recruitment rate was calculated using two metrics: the number of link clicks and number of completers per campaign. Using both metrics provided an understanding of whether the participants who clicked on our advertisement were doing so out of curiosity or whether they truly intended to participate in the study. The detailed breakdown provided by Facebook mobile allowed us to differentiate the reach and number of link clicks obtained through each Facebook and Instagram campaign, whereas Twitter gave this information directly. Chi-square tests were used to compare the demographic profile of individuals recruited via social media versus traditional means and to compare the profiles of individuals who were recruited across the 3 social media platforms.

#### Cost-effectiveness

Cost-effectiveness was measured using (1) cost-per-click and (2) cost-per-completer (Textbox 1). It was not possible to differentiate cost data between Facebook and Instagram; thus, data for these platforms were analyzed together.

### Ethics Approval

Template study advertisements were approved by the University of Calgary’s Conjoint Research Ethics Board (REB 20-1635) to ensure that they would not mislead participants regarding the purpose of the study.

## Results

### Recruitment Results

Between September 2021 and April 2022, a total of 324 participants enrolled in the ongoing P3 Cohort Study and completed the baseline survey across 13 separate advertisement campaigns. For Facebook and Instagram, 11 (85%) of the 13 campaigns were paid, and for Twitter, 5 (38%) of the 13 campaigns were paid. We reported the results of these paid campaigns. Of the 324 participants recruited, 153 (47%) heard about the study through Facebook, 79 (24%) through Instagram, 10 (3%) through Twitter, and 82 (25%) from other sources (eg, traditional methods including postcards and posters). Our paid advertisements reached 159,778 social media users that translated into 2390 link clicks.

Throughout our recruitment efforts, we encountered several issues regarding fraudulent responses. Of the 2390 link clicks, we initially had 1572 consents; however, upon further inspection, 1220 (78%) of these were deemed fraudulent, as indicated by made-up names and email addresses, as well as IP addresses outside the Calgary Zone of Alberta Health Services. Furthermore, out of 561 baseline surveys, 237 (42%) were deemed fraudulent because of inconsistencies in survey answers (eg, gestation age not matching the due date), nonsensical email addresses (eg, the email address primarily consisting of numbers or inconsistencies between a participant’s name and the name in their email address), and phone numbers and IP addresses outside of Calgary. These issues were most salient during our pilot campaign (labeled campaign 0). As such, we decided to stop mentioning the incentive in our caption and discontinued the paid advertisement to stop the circulation on social media. Subsequently, we implemented new security measures, including logic checks, monitoring for fake email addresses and duplicate IP addresses, and changing the landing page such that the website did not link directly to the consent form*.* Instead, when participants clicked on the link to join our study, they were required to answer some screening questions and were only then sent the consent form manually by the study team if the participant responses seemed legitimate. Screening questions asked for participant contact information (name, email address, and phone number) and basic demographic information to confirm eligibility (age, pregnancy status, gestational age, and place of residence). Members of the research team reviewed the responses and contacted participants if they were deemed eligible. These new measures led to an appreciable decrease in the rate of fraudulent responses; however, throughout the remainder of the recruitment period selected for this study, the team had to be diligent in monitoring survey response rates.

### Recruitment Rate per Platform

#### Facebook

##### Link Clicks

Our paid Facebook advertisements reached 124,515 users through 11 paid campaigns, which translated into 1916 link clicks, resulting in a click-through-rate (CTR) of 1.54% (1916/12,451) and a completion to click ratio of 7.99% (153/1916; Table 2). Campaign 6 was the most successful in generating link clicks on Facebook. Campaigns 8 to 10, which mentioned the incentive directly in the image of the advertisement, also generated much traffic to our study website. Campaign 4, the prematurity awareness advertisement, and campaign 12, which was discontinued after only a day of recruitment owing to fraudulent responses, were the least successful in generating link clicks.

**Table 2:** Recruitment rates for paid advertisements on Facebook, Instagram, and Twitter.

Campaign	Facebook			Instagram				Twitter		
	Reach, n	Link clicks (CTR^a^), n (%)	Completed surveys, n (%^b^)	Reach, n	Link clicks (CTR), n (%)	Completed surveys, n (%^b^)	Reach, n		Link clicks (CTR), n (%)	Completed surveys, n (%^b^)
0	—^c^	—	2	—	—	1	—		—	5
1	—	—	—	—	—	—	—		—	—
2	8698	134 (1.54)	10 (7.46)	146	2 (1.37)	1 (50)	549		3 (0.54)	—
3	6945	220 (3.16)	2 (0.91)	—	—	—	929		6 (0.65)	—
4	5313	50 (0.94)	8 (16)	2645	12 (0.45)	1 (8.33)	1798		12 (0.67)	1 (8.33)
5	10,956	167 (1.52)	14 (8.38)	288	8 (2.78)	1 (12.5)	7639		17 (0.23)	2 (11.76)
6	27,732	378 (1.36)	25 (6.61)	3416	85 (2.49)	9 (10.59)	4584		24 (0.52)	2 (8.33)
7	9016	89 (0.99)	4 (4.49)	864	15 (1.74)	5 (33.33)	—^d^		—^d^	—
8	8395	149 (1.77)	21 (14.09)	1879	69 (3.67)	14 (20.29)	—^d^		—^d^	—
9	12,147	274 (2.26)	23 (8.39)	1532	38 (2.48)	7 (18.42)	—^d^		—^d^	—
10	20,800	272 (1.31)	23 (8.46)	3480	62 (1.78)	16 (25.81)	—^d^		—^d^	—
11	12,599	154 (1.22)	6 (3.9)	5012	115 (2.29)	16 (13.91)	—^d^		—^d^	—
12	1914	29 (1.52)	15 (51.72)	502	12 (2.39)	1 (8.33)	—^d^		—^d^	—
Total	124,515	1916 (1.54)	153 (7.99)	19,764	418 (2.11)	79 (18.9)	15,499		62 (0.4)	10 (16.13)

^a^CTR: click-through-rate.

^b^Percentage refers to the ratio of the number of individuals who completed the baseline survey divided by the number of individuals who clicked on the advertisement (completer-to-click ratio).

^c^Data on these metrics were unavailable.

^d^Campaigns 7 to 12 were unpaid on Twitter; therefore, no information on reach or link clicks was collected.

##### Completion

Campaign 6, the advertisement with the highest budget, led to the most number of completed baseline surveys (25/153, 16.3%), whereas campaigns 8 and 9, which were the first to introduce the study’s incentive into the advertisement’s image and caption, were also highly successful in terms of recruitment (Table 2). Campaigns with lower budgets and duration (campaigns 3 and 7) led to the fewest number (2/153, 1.3%, and 4/153, 2.6%, respectively) of completed baseline surveys.

#### Instagram

##### Link Clicks

Our paid Instagram advertisements reached 19,764 users through the 11 paid campaigns, which translated into 418 link clicks, resulting in a CTR of 2.11% (418/19,764) and completion to click ratio of 18.9% (79/418; Table 2). As with Facebook advertisements, campaigns 6 and 8 to 10 were also the most successful in generating link clicks.

##### Completion

Campaigns 6, 10, and 11 led to the highest number of completed surveys (9/79, 11.4%; 16/79, 20.3%; and 16/79, 20.3%, respectively), on Instagram (Table 2). However, campaigns 2, 3, 4, 5, and 12 led to only one or no completed baseline surveys.

#### Twitter

##### Link Clicks

Our paid Twitter advertisements reached 15,499 users through the 5 paid campaigns, which translated into 62 link clicks, resulting in a CTR of 0.4% (62/15,499) and completion to click ratio of 16.13% (10/62; Table 2).

##### Completion

Although there was a higher proportion (10/62, 16.13%) of completers on Twitter compared with Facebook (153/1,916, 7.99%), the overall yield of the advertisements was low. Therefore, paid Twitter advertisements were discontinued after campaign 5 because of the low recruitment rate.

### Cost-effectiveness

#### Facebook and Instagram

The total cost for Facebook and Instagram advertisements was CAD $1430 (US $1859) throughout the 11 paid campaigns. Cost-per-click for Facebook and Instagram advertisements was consistently under CAD $1 (US $1.3) across campaigns, with an average cost-per-click of CAD $0.65 (US $0.84; SD $0.27; US $0.35) throughout the 11 campaigns. Campaign 3 saw the lowest cost-per-click at CAD $0.14 (US $0.18), whereas the highest cost-per-click was for campaigns 4 and 5, both at CAD $1 (US $1.3; Table 3).

The cost-per-completer on Facebook and Instagram remained <CAD $15 (US $19.5; Table 3). Campaign 12, which mentioned the incentive and had the second highest budget per day, saw the lowest cost-per-completer at CAD $1.44 (US $1.87) per participant. Overall, the average cost-per-completer was CAD $7.89 (US $10.25; SD $4.08, US $5.30).

**Table 3:** Cost-per-click and cost-per-completer for Facebook and Instagram (combined) and Twitter.

Campaign	Facebook and Instagram			Twitter	
	Cost-per-click, CAD $ (US$)	Cost-per-completer, CAD $ (US$)	Cost-per-click, CAD $ (US$)		Cost-per-completer, CAD $ (US$)
0^a^	—^b^	—^b^	—^b^		0.00^c^
1^a^	—^b^	—^b^	—^b^		—^b^
2	0.92 (1.19)	11.36 (14.7)	11.71 (15.22)		—^b^
3	0.14 (0.18)	15.00 (19.5)	4.18 (5.43)		—^b^
4	1.00 (1.3)	11.11 (14.44)	—^d^		0.00^c^
5	1.00 (1.3)	11.67 (15.08)	9.49 (12.33)		69.00 (89.7)
6	0.54 (0.70)	6.10 (7.93)	4.63 (6.01)		55.61 (72.2)
7	0.72 (0.93)	8.33 (10.8)	—^e^		—^b^
8	0.34 (0.44)	2.14 (2.78)	—^e^		—^b^
9	0.40 (0.52)	4.17 (5.41)	—^e^		—^b^
10	0.75 (0.97)	6.41 (5.11)	—^e^		—^b^
11	0.74 (0.96)	9.08 (11.8)	—^e^		—^b^
12	0.57 (0.74)	1.44 (1.87)	—^e^		—^b^

^a^Campaigns 0 and 1 were unpaid across all platforms.

^b^Data not available.

^c^Twitter campaigns 0 and 4 recruited a participant despite not using any budget, so they were likely to have heard about the advertisement through organic methods.

^d^Twitter did not use any of our budget for campaign 4.

^e^Campaigns 7 to 12 were unpaid on Twitter.

#### Twitter

The total cost of Twitter advertisements throughout the 5 paid campaigns was CAD $299 (US $388.7). Depending on the campaign, only some (or sometimes none) of the prespecified budget was used by Twitter to display our advertisements.

Cost-per-click for the advertisements that used a partial or full amount of the prespecified budget ranged from CAD $4.18 (US $4,72; campaign 3) to CAD $11.71 (US $15.22; campaign 2), with an average cost-per-click of CAD $7.50 (US $9.75; SD $3.20, US $4.16; Table 3). Cost-per-completer for paid Twitter advertisements ranged from CAD $55.61 (US $72.29; campaign 6) to CAD $69 (US $89.7; campaign 5).

### Representativeness

The demographic profiles of individuals recruited via social media did not differ from those recruited through traditional means in terms of education, household income, marital status, immigration status, race, or age (Table 4). However, among individuals who were recruited via social media, those with educational backgrounds of university graduation and above were more likely to be recruited via Instagram or Twitter (*P*<.001) and those with a higher household income (above CAD $100,000/year; US $13,000) were also more likely to be recruited through Instagram or Twitter (*P*<.001).

**Table 4:** Demographic characteristics of the participants recruited through Facebook, Instagram, Twitter, and other sources (N=307)^a^.

Characteristics			Facebook, n (%)		Instagram, n (%)		Twitter, n (%)		Other sources, n (%)		*P* value				
											For differences between 3 social media sources			For differences between social media and other sources	
**Education, (n=306^a^)**												<.001			.53
	Did not graduate university	26 (18)		7 (10)		0 (0)		9 (11)							
	Graduated university	92 (64)		44 (60)		2 (20)		47 (59)							
	Graduated school	25 (17.48)		22 (30)		8 (80)		24 (30)							
**Income in CAD $, (n=303^a^)**												<.001			.54
	<99,999 (US $129,998)	49 (35)		10 (14)		0 (0)		24 (30)							
	≥99,999 (US $129,998)	91 (65)		63 (86)		10 (100)		56 (70)							
**Marital status, (n=307^a^)**												.57			.34
	Married or common law	132 (92.31)		69 (95)		10 (100)		78 (96)							
	Other	11 (7.69)		4 (5)		0 (0)		3 (4)							
**Country of origin, (n=307^a^)**												.75			.20
	Canada	121 (85)		60 (82)		9 (100)		63 (78)							
	Other	22 (15)		13 (18)		1 (10)		18 (22)							
**Race, (n=306^a^)**												.17			.36
	White	107 (75)		53 (73)		10 (100)		57 (70)							
	Other	35 (25)		20 (27)		0 (0)		24 (30)							
**Age in years, (n=300^a^)**												.31			.64
	<35	100 (71)		44 (61)		6 (60)		54 (70)							
	≥35	41 (29)		28 (39)		4 (40)		23 (30)							

^a^Owing to item nonresponse, the total of the n values does not add up to 324.

## Discussion

### Principal Findings

Using social media as a recruitment strategy proved to be an effective method to reach and recruit a sample of pregnant women in our longitudinal study although the recruitment rate and cost-effectiveness did vary by platform. Facebook and Instagram were highly effective in generating traffic in our study survey. The CTR of 1.54% (1916/124,515) for Facebook and 2.11% (418/19,764) for Instagram were consistent with previous literature on this topic, with most studies finding a CTR of approximately 2% for Facebook advertisements [1,2,8,9]. The higher CTR on Instagram can likely be attributed to the fact that Instagram is more commonly used by our target demographic (especially among women aged 18-29 years [10]), while the success garnered by Facebook advertisements is likely related to the regular use of this platform by pregnant women to connect with other pregnant women and to find answers to pregnancy- or parenting-related questions [1,5,11]. Twitter was much less effective in generating traffic to our website and recruiting participants, despite being commonly used by younger and middle-aged women [10]. A possible explanation is that the Twitter algorithm is less effective at targeting and reaching the population of interest. Our Twitter account was contacted mainly by other researchers as opposed to accounts related to our target population.

The campaigns with the most success in terms of link clicks and completed surveys were those with either a high budget (campaign 6) or those that mentioned the study’s incentive directly in the image of the advertisement (campaigns 8-11). Campaign 12 was an exception because the advertisement had to be discontinued after only a day to a high influx of fraudulent responses, thus generating fewer link clicks. It is important to note that although campaign 3 had a high number of link clicks even with a low budget, the advertisement was mistakenly targeted to all of Canada, likely resulting in many link clicks and few completed surveys. Interestingly, campaign 4 was less successful in terms of generating link clicks and completed surveys, demonstrating that standard advertisements were more successful than themed ones. Advertisements posted for a shorter duration (3 days rather than 5 days) and without the incentive were also less successful in generating link clicks and completed surveys (campaign 7). The most successful advertisements contained a short title and description of the study, the logos of affiliated institutions, the incentive of the study, the study website link, and a relevant image or cartoon.

Paid Facebook and Instagram advertisements proved to be cost-effective, with an average cost-per-click of CAD $0.65 (US $0.84; SD $0.27; US $0.35) and an average cost-per-completer of CAD $7.89 (US $10.25; SD $4.08, US $5.30). This cost-per-completer was lower than what has been found in previous pregnancy studies that have used social media for recruitment [1,12-14]. The cost-per-completer in these studies ranged from CAD $14.63 (US $19.01) [14] to CAD $51.27 (US $66.65) [13]. The average cost-per-click of CAD $0.65 (US $0.84; SD $0.27, US $0.35) was similar to findings in other pregnancy studies [1,12]. Overall, Facebook and Instagram advertisements provided a cost-effective method to reach our target population, especially in comparison to the cost normally incurred by traditional methods where researchers must spend time and money designing, printing, and distributing posters and brochures [1,14]. An important consideration, however, is that it was quite time-consuming for our study team to monitor and sort through fraudulent participants. Paid advertisements on Twitter were less cost-effective than Facebook and Instagram advertisements, with an average cost-per-click of CAD $7.50 (US $9.75; SD $3.20, US $4.16) and cost-per-completer ranging from CAD $55.61 (US $72.2) to CAD $69 (US $89.7). Focusing on using organic (ie, unpaid) recruitment methods rather than paid advertisements on Twitter may help overcome some of the challenges we faced when using this platform.

Overall, the characteristics of the participants recruited in this study reflected those of participants in other studies that focused on pregnancy or other studies examining the effectiveness of social media recruitment [1,2,9,15,16]. Our sample mainly comprised highly educated and higher-income White women born in Canada. On the basis of the results of our analysis, the demographic characteristics of the participants recruited through social media did not differ significantly from those of participants recruited through traditional methods. Therefore, the use of social media for recruitment will not leave out important demographics that would otherwise have been obtained using only traditional methods. In addition, we found that all 3 social media platforms recruited participants with similar demographic characteristics; however, Facebook was more effective than Twitter and Instagram in recruiting individuals with lower education and income levels. This might be related to the fact that our Twitter page was primarily followed by researchers, although there is less obvious explanation for Instagram. Importantly, however, in our overall sample, individuals with lower levels of education and income remained underrepresented.

An increasing number of studies using social media for recruitment have reported issues regarding fraudulent responses. One study that used Facebook and Twitter for recruitment to examine patient perceptions of patient-provider communication in the ovarian cancer care setting found that most of their survey respondents were illegitimate [17]. They suggested indicators of low-quality data, including evidence of inattention (completing the survey in an unrealistic amount of time), duplicate or unusual responses to open-ended survey items, inconsistent responses to verifiable items (eg, the location of the survey respondent and the time zone not matching), and evidence of bot automation [17]. Similar indicators were used to flag fraudulent participants in this study. Studies suggest that web-based private servers and server farms may be at the root of this issue, as they allow one individual to complete many surveys simultaneously, each with a unique IP address, purely for financial gain [17,18]. Consequently, research funds meant to compensate legitimate study participants are used for bot responses [18]. As IP addresses are not tied to physical locations, but rather are assigned by internet service providers when users access the internet, they can be manipulated, and IP addresses alone cannot be used to identify real versus fraudulent responses. Many other strategies exist to mitigate the fraudulent responses, including lowering the value of the incentive, collecting verifiable information (phone number), sending each respondent a unique survey link, having items that can be compared for consistency, capturing time stamps for start and stop times, requiring open-text questions, or limiting the visibility of the advertisement on social media platforms [17-21]. In addition, having participants check a box stating that they understand that ineligible responses will not receive the incentive and that researchers may contact them by phone to confirm eligibility may drive illegitimate participants away from the study [18]. Ultimately, researchers must find a balance between making the study easy and convenient for legitimate respondents to participate and establishing sufficient security measures to preserve the integrity of the data [21]. Too many steps (ie, screening questions, CAPTCHA [Completely Automated Public Turing test to tell Computers and Humans Apart] tests, etc) may cause frustration among real participants and lead to a lower recruitment rate. More research is needed in this area to determine the best approach for mitigating fraudulent responses while maintaining the convenience and cost-effectiveness of social media recruitment.

### Strengths and Limitations

This study has both strengths and limitations. To the best of our knowledge, this is one of the first studies to directly compare the ability of multiple social media platforms to recruit participants. This provides valuable information that other researchers can use to help determine the best recruitment source for their needs and to accurately estimate their budget. This study also has several limitations. First, owing to the nature of our study design, we were unable to know where consenting participants heard about the study. This metric would have allowed us to determine whether participants who clicked on our website and consented followed through with participating in the study (or if they just clicked on the advertisement out of curiosity) based on which platform they heard about the study. In addition, we could not determine whether there was a major difference in the cost-per-consent and cost-per-completer. A solution to this would be to include a question asking where they heard about the study on the consent form as opposed to only on the baseline survey. Evaluation of social media as a recruitment method can be challenging owing to the ever-changing policies and algorithms, making it difficult to maintain consistency over a long recruitment period and to compare with other studies of a similar nature [2]. The potential for recall bias should also be considered, as participants may have seen the study advertisement in many places or forgotten how they heard about the study, thereby influencing our results. Similar to other prospective pregnancy cohort studies, this study underrepresented individuals with lower education, lower income, and racial minorities. This poses a problem in generalizing the findings of the broader cohort study to the general population. To counter this, studies have suggested targeting advertisements to lower-income postal codes or neighborhoods within a city or targeting advertisements to the interests of minority populations [22,23]. Future research should focus on these strategies. By focusing on social media strategies aimed at recruiting underrepresented populations, studies could identify common factors of pregnancy in these populations that make them susceptible to preterm birth. This could ultimately lead to more targeted interventions to reduce health disparities within the community. In addition, the advertisements in this study were specifically targeted at social media users with an interest in pregnancy and parenting (because the social media algorithm did not permit us to directly target advertisements to pregnant women). Therefore, our sample likely consists of individuals who either discuss their pregnancy openly on social media or individuals who like and follow pregnancy-related pages. Future research could investigate the effectiveness of generic advertisements (ie, advertisements targeted at all women) compared with more targeted advertisements such as those used in this study. Finally, the advertisements and content used to promote this study were targeted toward women; however, we recognize that not all individuals who are pregnant identify themselves as women. In addition, much of the content focused on the positive and exciting aspects of pregnancy; however, pregnancy and the transition to parenthood can be challenging. Future content can aim to overcome this by being more inclusive of different gender identities and experiences of pregnancy.

### Conclusions

In conclusion, the results of this study demonstrate that social media is a feasible and cost-effective way to reach and recruit pregnant women to a longitudinal study. Paid advertisements on Facebook and Instagram, specifically, were highly practical and cost-effective methods of reaching and recruiting participants. Researchers can turn to this work to gain an understanding of what to expect in terms of recruitment rate, cost, and representativeness when using social media to recruit pregnant women or other populations. However, researchers should be aware of the potential fraudulent responses and identify mitigation strategies for such issues. With ever-changing technology and the competitive nature of obtaining research funding, researchers should use social media to their advantage as an effective and low-cost means of recruitment. Ultimately, this work feeds into the broader P3 Cohort Study and is the first step toward gaining a better understanding of preterm births. As of April 2022, the P3 Cohort Study is ongoing, and, as such, the findings from this study will help inform our recruitment strategy going forward. Recruiting an appropriate number of participants is crucial to a study of this nature; therefore, the findings of this kind of work cannot be overlooked.

## Abbreviations

CTR: click-through-rate
